# Construction of a 5-gene prognostic signature based on oxidative stress related genes for predicting prognosis in osteosarcoma

**DOI:** 10.1371/journal.pone.0295364

**Published:** 2023-12-01

**Authors:** Xiaofang Hong, Ribin Fu

**Affiliations:** 1 Department of Stomatology, Zhongshan Hospital, Xiamen University, Xiamen, China; 2 Department of Joint Surgery and Sports Medicine, Zhongshan Hospital, Xiamen University, Xiamen, China; Affiliated Hospital of Nantong University, CHINA

## Abstract

**Background:**

The understanding of the complex biological scenario of osteosarcoma will open the way to identifying new strategies for its treatment. Oxidative stress is a cancer-related biological scenario. At present, it is not clear the oxidative stress genes in affecting the prognosis and progression of osteosarcoma, the underlying mechanism as well as their impact on the classification of osteosarcoma subtypes.

**Methods:**

We selected samples and sequencing data from TARGET data set and GSE21257 data set, and downloaded oxidative stress related-genes (OSRGs) from MsigDB. Univariate Cox analysis of OSRG was conducted using TARGET data, and the prognostic OSRG was screened to conduct unsupervised clustering analysis to identify the molecular subtypes of osteosarcoma. Through least absolute shrinkage and selection operator (LASSO) regression analysis and COX regression analysis of differentially expressed genes (DEGs) between subgroups, a risk assessment system for osteosarcoma was developed.

**Results:**

45 prognosis-related OSRGs genes were acquired, and two molecular subtypes of osteosarcoma were clustered. C2 cluster displayed prolonged overall survival (OS) accompanied with high degree of immune infiltration and enriched immune pathways. While cell cycle related pathways were enriched in C2 cluster. Based on DEGs between subgroups and Lasso analysis, 5 hub genes (ZYX, GJA5, GAL, GRAMD1B, and CKMT2) were screened to establish a robust prognostic risk model independent of clinicopathological features. High-risk group had more patients with cancer metastasis and death as well as C1 subtype with poor prognosis. Low-risk group exhibited favorable OS and high immune infiltration status. Additionally, the risk assessment system was optimized by building decision tree and nomogram.

**Conclusions:**

This study defined two molecular subtypes of osteosarcoma with different prognosis and tumor immune microenvironment status based on the expression of OSRGs, and provided a new risk assessment system for the prognosis of osteosarcoma.

## Introduction

Osteosarcoma as one the most frequently detected malignant tumors in bone system usually occurs in the metaphysis of long renal tubules, accounting for 0.2% of all solid malignant tumors, and it mainly threatens children and adolescents with exuberant bone growth, accounting for 3–6% of childhood cancers [1–4]. For high-grade osteosarcoma, the combination of surgery and chemotherapy is the first choice [5]. Despite treatment, 30% to 40% of patients will relapse within 2 to 3 years after treatment [6]. Worse, there is no standard treatment for patients with recurrence or metastasis, and the 5-year survival rate is only 20% [7–9]. Therefore, digging effective therapeutic biomarkers [10] for predicting treatment response, and monitoring indicators for early metastasis [11] to ameliorate the survival of osteosarcoma patients is urgent.

Oxidative stress is a cancer-related biological situation, which means that the intracellular reactive oxygen species (ROS) is high [12, 13]. Tumor development and anticancer treatment response involves the regulation of oxidative stress [14]. Previous research showed evidence of the relationship between some molecular markers related to oxidative stress and the progression and prognosis of cancer. In gastric cancer, 11 prognostic oxidative stress molecules (SERPINE1, CTLA4, HBB, F5, AGT, KIT, GPX3, GAD1, CYP19A1, BBC3 and NOX4) were involved in tumor progression [15]. To independently evaluate the prognosis of operable breast cancer, past study produced a systematic oxidative stress score based on five oxidative stress biomarkers (CRE, TBIL, LDH, BUN and ALB) [16]. A recently published paper also constructed a risk model with three genes related to oxidative stress (ERO1A, CDC25C and ITGB4), which is related to the clinical manifestations of patients with lung adenocarcinoma [17]. More importantly, numerous studies have disclosed that anticancer therapies containing immunotherapies through regulating ROS levels are prospective [18]. For example, monoamine oxidase A regulated tumor-associated macrophage polarization through upregulating oxidative stress to improve cancer immunotherapy [19]. Therefore, Leone and other researchers pointed out that according to different oxidative stress genes, tumors should be classified into subcategories to ensure precision medicine-based method development in certain subgroup of cancer patients [20]. In this point, exploring the studies on the biomarkers of ROS in osteosarcoma and their impact on immunotherapy may provide new therapeutic strategies.

In this study, our plan is to classify osteosarcoma into subclasses and construct a risk assessment system by oxidative stress-related genes and to study the biological differences such as tumor immune microenvironment (TIME) and pathway enrichment analysis between subclasses and risk groups. We also aim to design a nomogram for promoting personalized osteosarcoma treatment.

## Materials and methods

### Collection of osteosarcoma data

Osteosarcoma patients’ clinical follow-up information and RNA-seq data were collected from the database of Therapeutically Applicable Research to Generate Effective Treatments (TARGET, https://ocg.cancer.gov/programs/target) and processed using R packages “TCGAbiolinks” [21]. Only the genes encoded in the database and expressed in at least 50% of the samples were retained, with a total of 84 samples. Osteosarcoma sample data in the GSE21257 dataset was achieved from Gene Expression Omnibus, 53 of them were included in this study.

### Clustering for the oxidative stress related-gene

“GOBP_RESPONSE_TO_OXIDATIVE_STRESS” gene set was downloaded from Molecular Signatures Database (MsigDB), which covered 356 oxidative stress related-genes (OSRGs). OSRGs associated with osteosarcoma prognosis were screened and entered into the R package “ConsensusClusterPlus” [22] by performing univariate COX regression analysis of OSRGs in TARGET using the R “Survival” package [23]. In the process of unsupervised hierarchical clustering analysis, the algorithm we set was “hc”, the measurement distance was “pearson”, the bootstarps was 500, and the cluster range was 2–10.

### Conclusion of tumor immune microenvironment (TIME)

Different methods were implemented to evaluate the indicators in the TIME. ESTIMATE method [24] applied immune score and stromal score and ESTIMATE score as the criteria for evaluating immune components, matrix content and tumor purity. The abundance of infiltrating immune cells in TIME was quantified by Microenvironment Cell Populations-counter (MCP-counter) [25] and ssGSEA [26] based on 28 immune cell subsets [27], TIMER [28], EPIC [29], respectively.

### Prediction of the response to PD-1 therapy

T cell inflammatory microenvironment characterized by cytotoxic effector molecules, antigen presentation, active IFN- γ signal transduction, T cell active cytokines is a common feature of tumor biology in response to PD-1 checkpoint blocking [30]. In this study, IFN- γ scores and immunocyte infiltrated cytolytic (CYT) scoreswere calculated according to type 1 helper T (Th1) / IFN- γ gene signatures [31] and the expression of PRF1 and GZMA [32], respectively. Ayers et al. proposed T cell-inflamed gene expression profile (GEP) score, which predicts the potential clinical response of samples to PD-1 checkpoint blockade based on the T-cell-inflamed GEP of 18 genes [30]. Based on this, this study compared the performance of T cell inflamed GEP score between the clusters divided by clustering analysis.

### Functional annotation

We extracted the GSEA hallmark set “h.all.v7.5.1.entrez.gmt” from MSigDB. The GSEA software tool [33] was adopted to calculate the pathway activity of different subgroups, and compared them between groups. The differential enrichment pathway between subgroups was determined by setting the threshold value within false discovery rate (FDR) < 0.05.

### Differential expression analysis and establishment of a risk assessment system

LIMMA package [34] of R was adopted to identify OSRGs differentially expressed genes (DEGs) between relevant subtypes [8] with the threshold of FDR < 0.05 & | |fold change (FC)| > 1.5. The DEGs was analyzed by univariate COX regression, and genes showing potential prognostic value were screened with p < 0.001 as the threshold. LASSO Cox regression analysis in R with 10-fold cross-validation was performed to identify prognostic DEGs. To obtain the most concise risk assessment system, stepwise multivariate COX regression analysis was adopted to delete the variables that had little contribution to osteosarcoma prognosis by LASSO regression with “glmnet” package [35], and by combining the variables retained by multistep screening, a risk assessment system was established. Each sample in TARGET and GSE21257 datasets was assigned with a risk score by the risk assessment system and then normalized by Z score. Samples with Z score > 0 were divided into the high risk group, while Z score < 0 was divided into low risk group. The Kaplan-Meier curve (K-M curve) and logarithmic rank test were carried out to evaluate the survival difference between groups. The receiver operating characteristic [20] curves were subjected to describe the prognosis value of risk assessment system using the “timeROC” package [36].

### Construction of a nomogram and a decision tree

The optimization model based on risk assessment system was constructed, including a decision tree and a nomogram. The sample clinical features (age, gender, metastatic) given in TARGET and the risk assessment system were selected as input features in “rpart” to recursive partition and build a decision tree model. To screen independent prognostic factors with a cut value of p < 0.05, we performed univariate and multivariate COX regression analysis before constructing nomogram. The independent prognostic factors were incorporated into the “RMS” package of R software [37] to construct nomogram, and the performance of nomogram model was evaluated based on calibration curve, decision curve analysis (DCA) and ROC curve.

### Statistical analysis

The analysis and calculation of all data and the visualization of graph were performed using R software. Student T test was employed for comparisons between two groups, the statistical correlation of parametric variables was assessed by Pearson correlation. K-M curve was drawn by “survminer” package [38] and logarithmic rank test was adopted to analyze how overall survival (OS) was related to the risk score. Results with a p < 0.05 were considered as a statistical significance if not otherwise specified.

## Results

### Identification of two molecular subtypes of osteosarcoma with different clinical characteristics based on OSRGs unsupervised clustering

**Fig 1:**
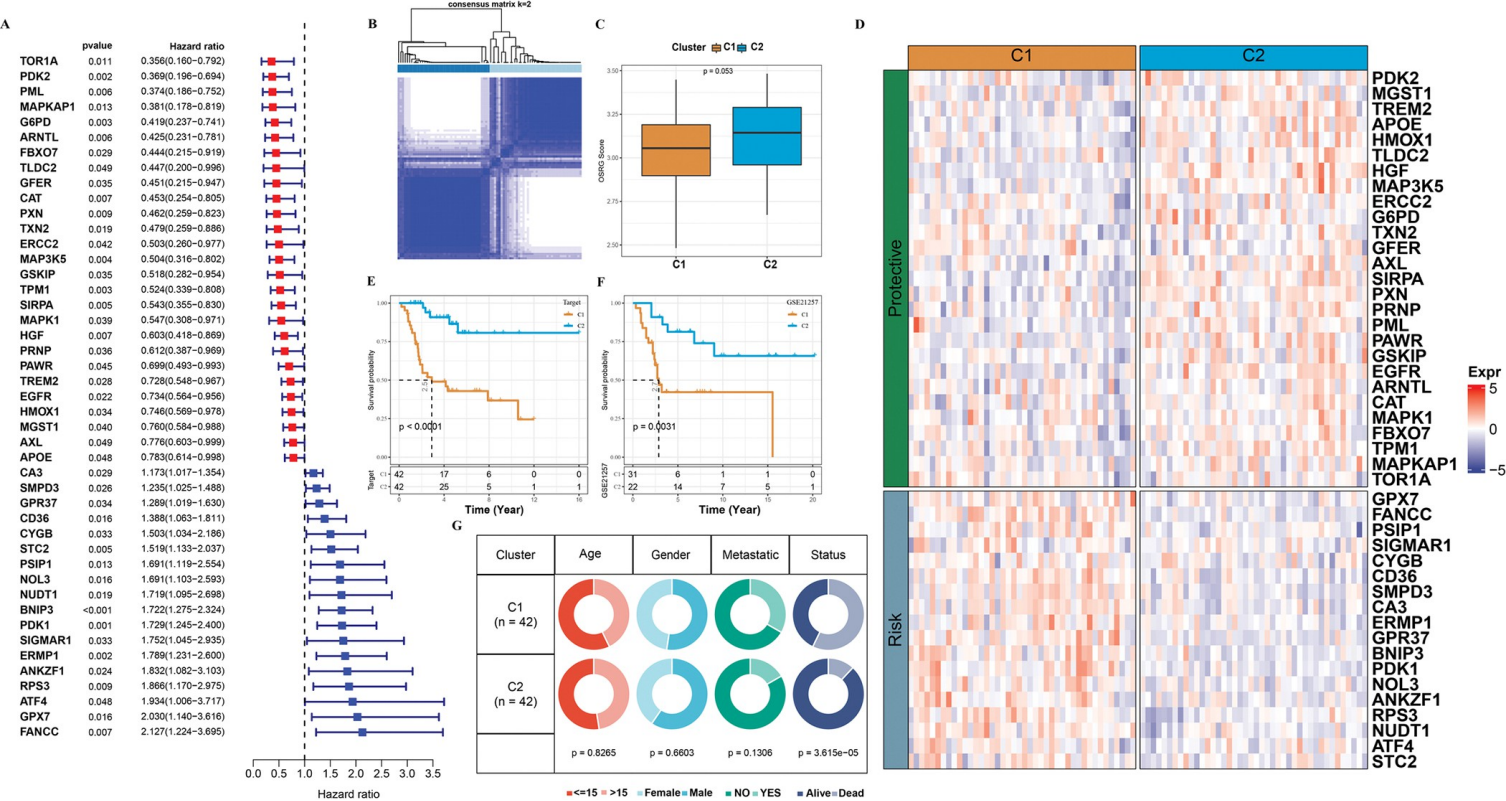
Identification of two molecular subtypes of osteosarcoma with different clinical characteristics based on OSRGs unsupervised clustering. A: Univariate COX regression forest plots for 45 OSRGs in the TARGET dataset. B: Sample clustering heatmap for consensus k = 2 in TARGET cohort. C: The box chart shows the OSRGs score difference between two subgroups in TARGET dataset. D: Expression heatmap of OSRGs associated with osteosarcoma prognosis in distinct subgroups of TARGET. E: K-M curves and survival differences for two clusters in the TARGET cohort. F: Prognostic differences between the two subgroups in the GSE21257 cohort. G: Comparison of clinical features of two subgroups in TARGET.

According to the OSRGs expression level given in TARGET, univariate COX regression analysis was conducted through the coxph function in R. The results showed that 45 OSRGs had statistical significance in the prediction of osteosarcoma. The hazard ratio [21] of 27 of them were < 1, was protective factors for osteosarcoma prognosis, and 18 of them were poor prognostic factors for osteosarcoma, with HR > 1 (**Fig 1A**). According to hierarchical clustering analysis for the expression of 45 OSRGs, we identified two molecular subtypes of osteosarcoma, C1 and C2 (**Fig 1B**). The OSRGs score of each sample in TARGET was calculated by ssGSEA and compared between the two subgroups. We found that the OSRGs score of C2 was higher than C1, and the difference of OSRGs score between the two groups was at the borderline significant (**Fig 1C**). Meanwhile, the expression of 45 OSRGs in two subgroups was shown directly in the form of heatmap (**Fig 1D**). It was found that in C1, 18 risk OSRGs were generally highly expressed and 27 protective OSRGs were generally under-expressed. However, in C2, the expression of 45 OSRGs showed an opposite trend than that in C1. The prognostic analysis of C1 and C2 subtypes in TARGET and GSE21257 demonstrated a significantly worse prognosis of C1 than C2 (**Fig 1E and 1F**). In term of age, gender and metastatic, the distribution of the two subgroups did not show statistical significance, although difference existed. The proportion of dead samples in C1 subgroup with poor prognosis was more than C2 (**Fig 1G**). These results together suggested that prognostic related OSRGs indeed could classify osteosarcoma patients into subcategories and effectively evaluate the prognostic differences between molecular subtypes.

### The two subgroups of osteosarcoma showed different TIME and the response to PD-1 therapy

**Fig 2:**
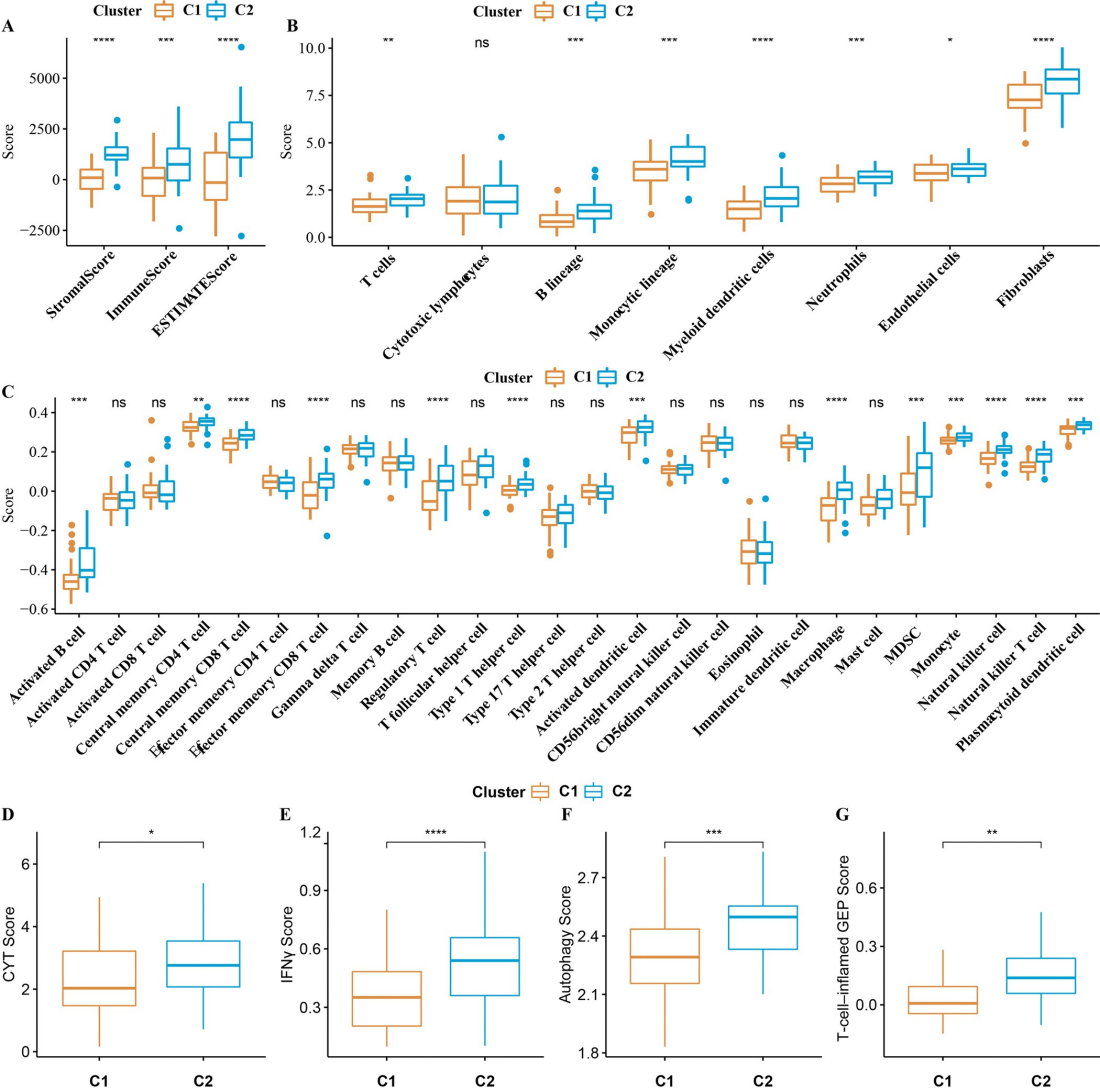
The two subgroups of osteosarcoma showed different TIME and the response to anti-programmed death-1 (PD-1) therapy. A: The difference of immune score, stromal score and ESTIMATE score between C1 and C2 calculated by ESTIMATE. B: The abundance difference of 10 immune cells in C1 and C2 quantified by MCP-counter. C: Differences in infiltrating immune cell enrichment scores calculated by ssGSEA between C1 and C2. D-G: Differences in CYT score, IFN- γ score, T cell inflamed GEP score and autophagy score between C1 and C2. (T-test, significance of difference is marked with *,* *P* <0.05; ** *P* <0.01; *** *P* <0.001; **** *P* <0.0001).

We explored the TIME of two subgroups defined by OSRGs in TARGET cohort. ESTIMATE showed significant differences between C1 and C2 in terms of immune score and stromal score and ESTIMATE score. Compared with C1, all three types of TIME score were significantly up-regulated in C2 (**Fig 2A**). MCP-counter quantified the abundance of 10 immune cells in C1 and C2, and the abundance of 9 immune cells showed significant difference between C1 and C2, and both were significantly higher in C2 (**Fig 2B**). Similarly, among the infiltrating immune cell enrichment scores obtained by ssGSEA, the scores of 13 kinds of immune cells (myeloid-derived suppressor cells (MDSC), central memory CD8 T cell, effector memory CD8 T cell, natural killer T cell, activated B cell, regulatory T cell, central memory CD4 T cell, type 1 T helper cell, natural killer T cell, macrophage, monocyte, activated dendritic cell, plasmacytoid dendritic cell) were higher than that in C1 (**Fig 2C**). Considering that T cell inflammatory microenvironment characterized by T cell active cytokines, cytotoxic effector molecules, and active IFN- γ signal transduction is an important part of blocking reactive immune microenvironment at PD-1 checkpoint. Here, the CYT score, IFN-γ score, T cell inflamed GEP score and autophagy score of C1 and C2 were analyzed, the scores of these T-cell inflammatory microenvironments in C2 were significantly higher than C1 (**Fig 2D–2G**). The results showed that C2 with high degree of immune infiltration was more likely to respond to PD-1 checkpoint blocking than C1.

### Differences in signal pathways of two subgroups enrichment in osteosarcoma

**Fig 3:**
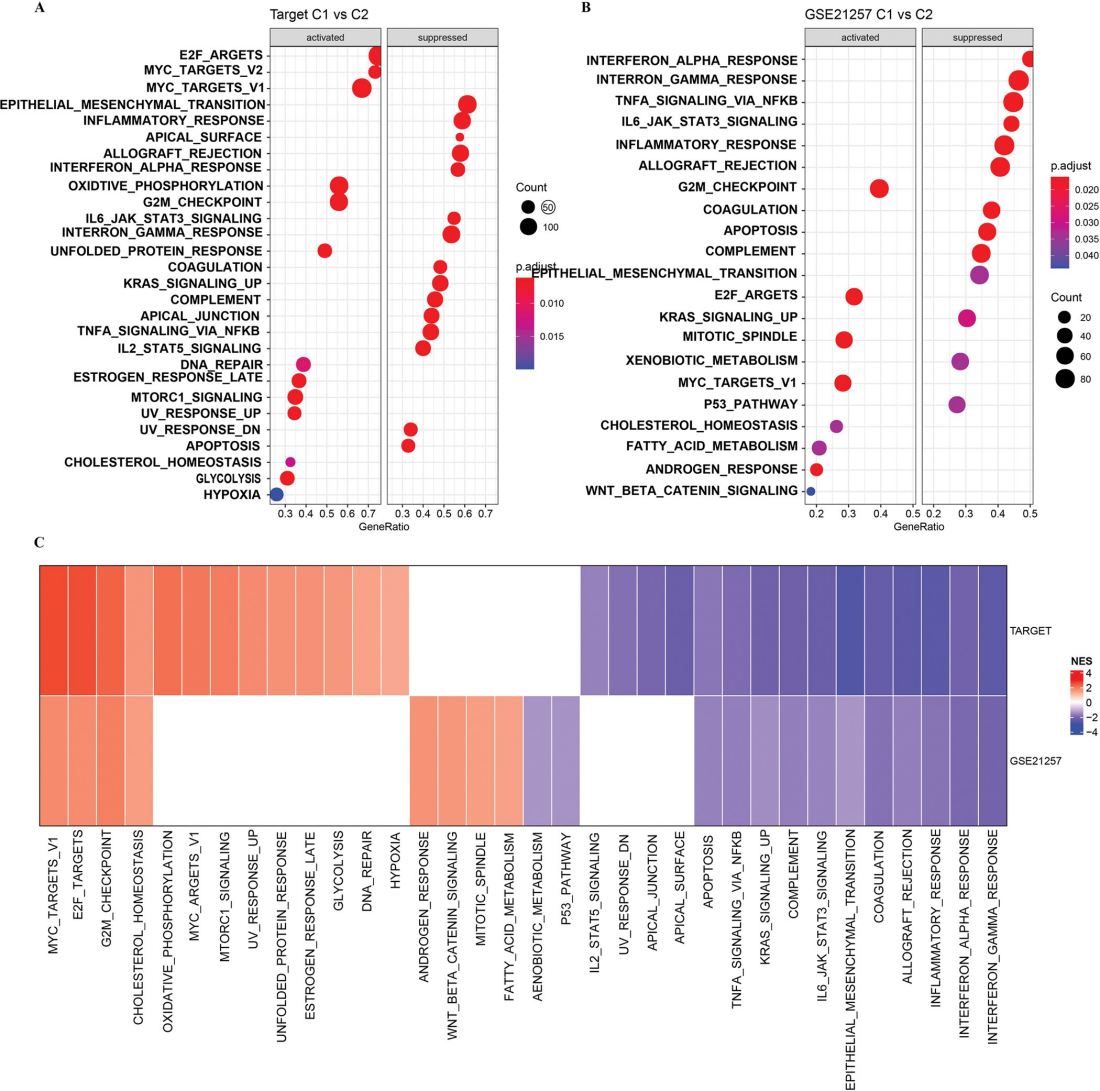
Differences in signal pathways of two subgroups enrichment in osteosarcoma. A: GSEA analyzed the signaling pathways that showed significant differences between C1 and C2 in TARGET data sets. The left box represents the pathway that is significantly activated in C1 compared to C2, and the right box represents the pathway that is significantly inhibited in C1 vs C2. B: GSEA analyzed the signal pathways that showed significant differences between C1 and C2 in GSE21257 datasets. C: C1 Comparison of significantly activated and significantly inhibited pathways in C1 compared to C2 between TARGET and GSE21257 datasets.

The above results provided evidence for the TIME heterogeneity of two subgroups in osteosarcoma. Then, we studied the mediated functional differences between the two molecular subtypes. GSEA identified several signal pathways that showed significant differences between C1 and C2. In the TARGET dataset, compared with C2, 13 pathways in C1 were activated and 15 pathways were inhibited. Most of the pathways leading to cell-cycle regulation (including MYC targets, E2F targets, DNA repair, G2M checkpoint) were included in the list of activated pathways. The suppressed pathway was mainly involved in immune-associated regulation (including allograft rejection, inflammatory response, complement, and interferon alpha response), epithelial-mesenchymal transition and apoptosis (**Fig 3A**). Similarly, in the GSE21257 dataset, the pathways that were significantly activated and inhibited in C1 relative to C2 showed the same state as in TARGET (**Fig 3B**). In the two datasets, compared with C2, the pathways significantly activated and inhibited by C1 overlap a lot, and the biological functions mediated by C1 are surprisingly consistent as a whole (**Fig 3C**).

### Establishment and verification of risk assessment system

**Fig 4:**
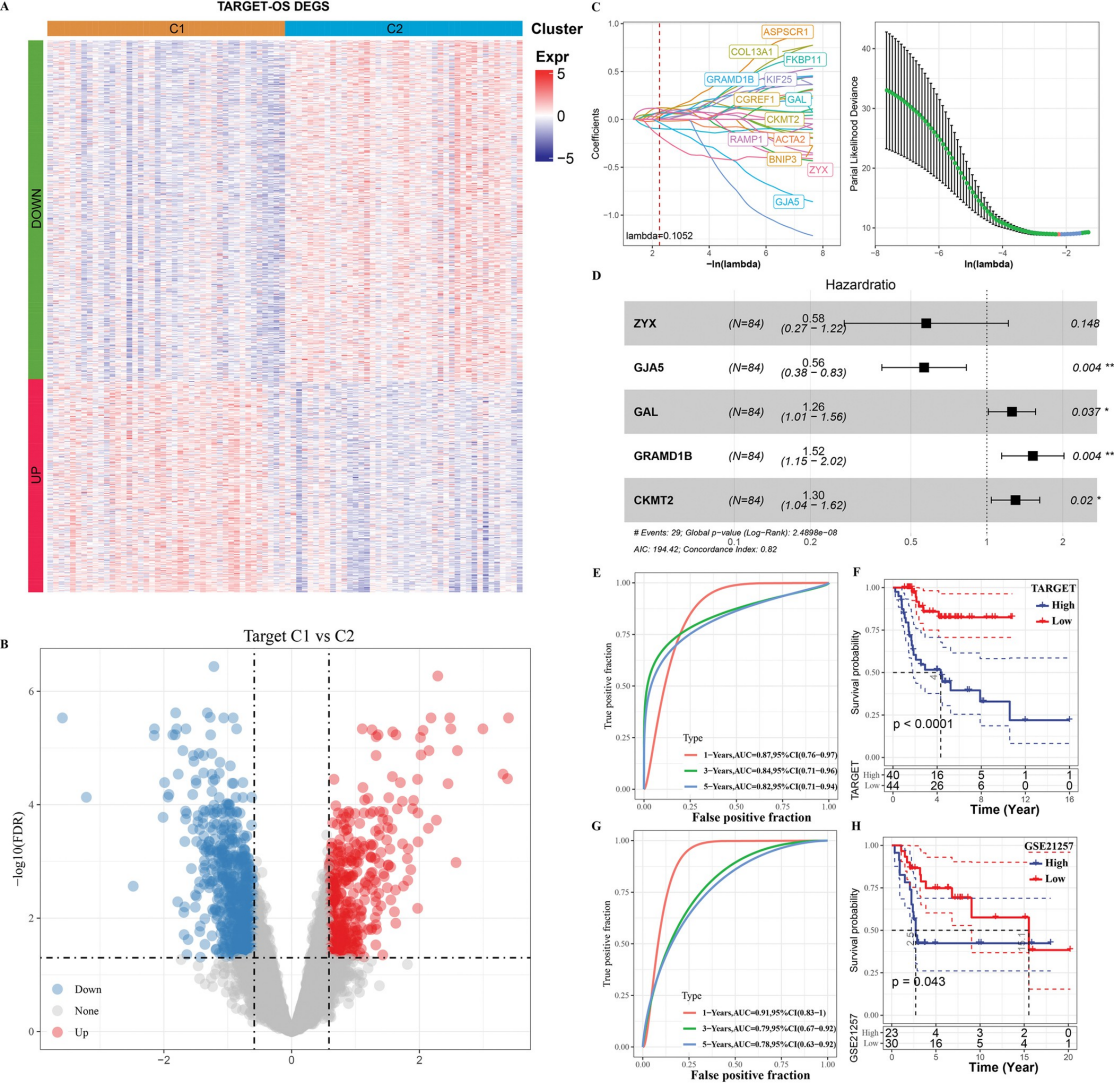
Establishment and verification of risk assessment system. A: The volcano diagram shows the DEGs of C1 compared to C2 in TARGET. B: Heatmap shows the expression pattern of DEGs in C1 and C2 of TARGET. C: Lasso regression process of 30 genes with potential prognostic ability. D: Multivariate COX analysis based on stepwise regression was adopted to identify the key genes with the greatest prognostic impact on osteosarcoma. E: ROC curve assessed the accuracy of the risk assessment system in predicting the prognosis of osteosarcoma patients in the TARGET cohort. F: Survival K–M curves for the patients with diverse risk score in the TARGET cohort. G: ROC curve assessed the accuracy of the risk assessment system in predicting the prognosis of osteosarcoma patients in the GSE21257 cohort. H: Survival K–M curves for the patients with diverse risk score in the GSE21257 cohort.

Due to the heterogeneity of TIME and mediated biological pathways between C1 and C2, the DEGs between the two molecular subtypes was screened by difference analysis for further analysis. We identified 1005 DEGs between C1 and C2 in TARGET (**Fig 4A**). Compared with C2, 621 DEGs were down-regulated and 384 DEGs were up-regulated in C1 (**Fig 4B**). Univariate COX regression analysis screened 30 potential prognostic genes out of 1005 DEGs with p < 0.001 as the threshold. LASSO regression eliminated the multicollinearity and screened 16 genes (**Fig 4C**). Multivariate COX analysis based on stepwise regression finally identified the five genes that had the greatest impact on the prognosis of osteosarcoma, which were ZYX, GJA5, GAL, GRAMD1B and CKMT2 (**Fig 4D**). The established risk assessment system was: risk score = -0.552×ZYX-0.572×GJA5 +0.229×GAL +0.42×GRAMD1B+0.262×CKMT2. TARGET was used as the training set, and the ROC curve was plotted to evaluate the risk assessment system’s accuracy. In the first year, the AUC of ROC curve was 0.87, 0.84, 0.82 at 1 year, 3 years and 5 years, respectively (**Fig 4E**). The high-risk group manifested significantly worse survival rate than low-risk group of patients at different time points (**Fig 4F**). We tested the performance of the risk assessment system we created in the GSE21257 dataset. In this verification set, we also detect that the OS of the high-risk group was significantly lower than that of the low-risk group (**Fig 4H**). The AUC of the ROC curve reached 0.91 (in 1 year) and 0.78 in the 5 years (**Fig 4G**). It means that the risk evaluation system we created had a relatively stable and good performance in predicting the prognosis of osteosarcoma in both test set and verification set.

### Clinical correlation of risk assessment system

**Fig 5:**
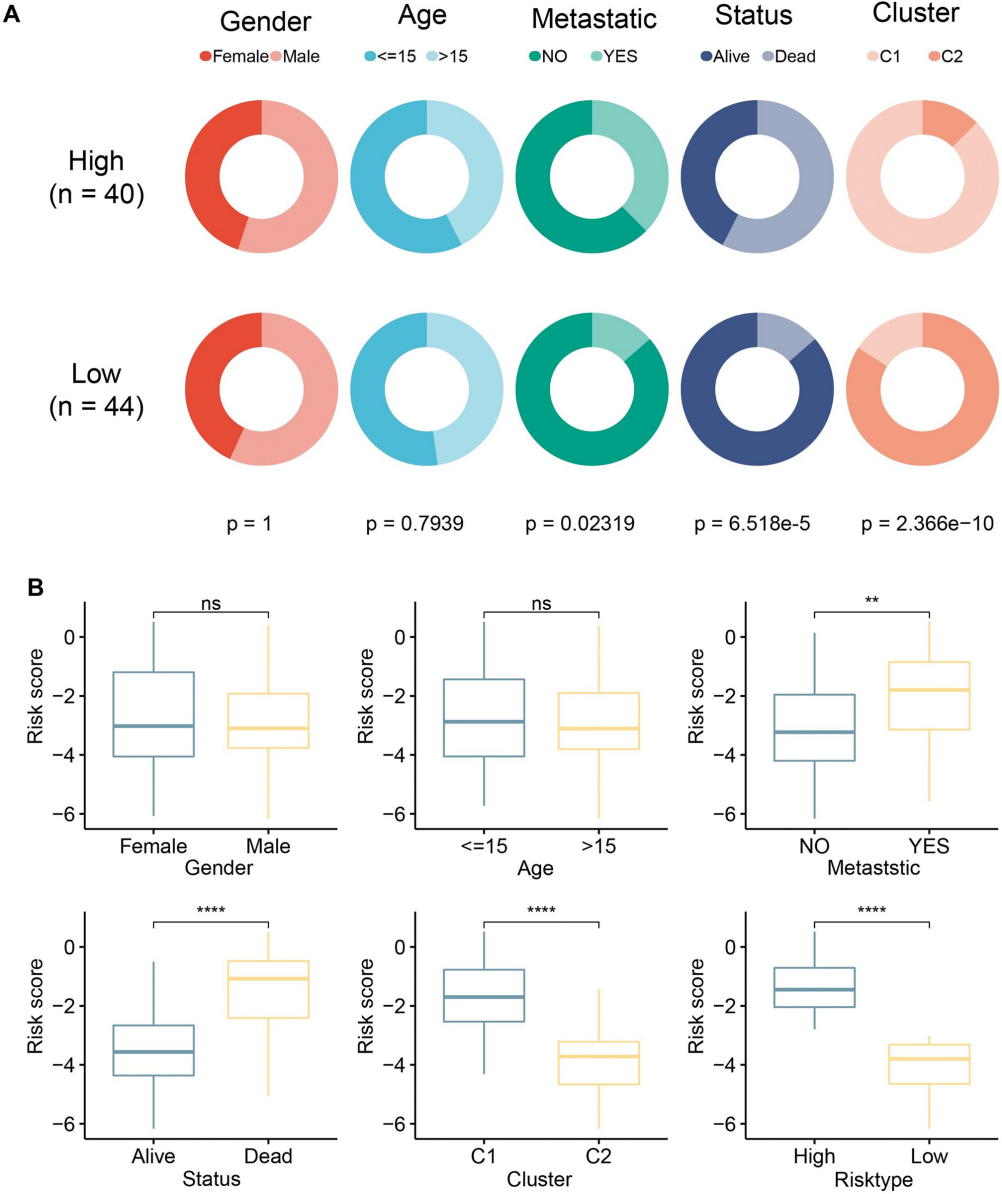
Clinical correlation of risk assessment system. A: Differences in the distribution of clinical features (chisq.test). B: The risk score calculated by the risk assessment system is compared between hierarchical subgroups according to gender, age, metastatic, status, and molecular subtypes. Ns: no significant difference; *: p<0.05, **: p<0.01, ***: p<0.001, ****: p<0.0001.

In the TARGET cohort, we evaluated the clinical relevance of the risk assessment system. Firstly, the differences of clinical manifestations were analyzed in the two risk groups. In the distribution of gender and age between high-risk group and low-risk group, no significant difference has been detected. There were significant differences in the degree of metastatic, survival state and distribution of molecular subtypes between the two groups (**Fig 5A**). No significant difference was found in risk score between subgroups stratified by gender (female & male) and age (< = 15 & > 15). Risk score was significantly correlated with metastatic degree, survival status, molecular subtypes and risk type. Samples with metastasis, death, C1, and metastasis and high risk exhibited significantly up-regulated risk scores relative to the clinical states that were in opposition to them (**Fig 5B**).

### TIME and biological processes of risk score calculated by the risk assessment system

**Fig 6:**
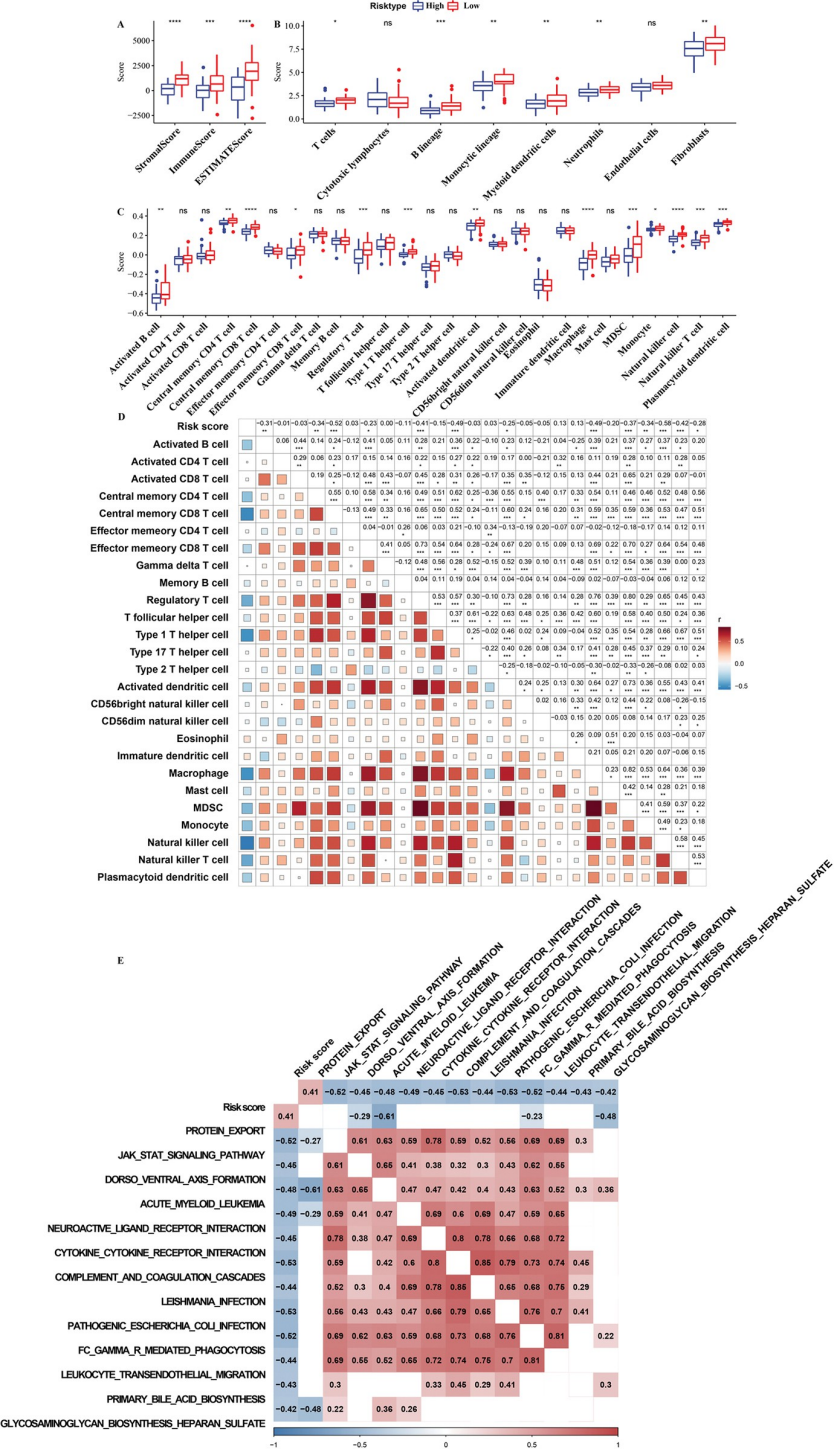
TIME and biological processes of risk score calculated by the risk assessment system. A: The difference of stromal score and immune score and ESTIMATE score between high-risk group and low-risk group. B: The level of immune cells calculated by MCP-counter was in the high-risk group and the low-risk group. C: Comparison of tumor immune infiltrating cell score calculated by ssGSEA between high-risk and low-risk groups. D: The correlation between score and risk score of tumor immune infiltrating cells was calculated by Pearson correlation test. E: Display of signaling pathways significantly related to risk score. Ns: no significant difference; *: p<0.05, **: p<0.01, ***: p<0.001, ****: p<0.0001.

We studied the TIME of risk score calculated by the risk assessment system. ESTIMATE analysis showed that the high-risk group was significantly correlated with lower stromal score and immune score and ESTIMATE score (**Fig 6A**). According to the relationship between the level of immune cells calculated by MCP-counter and ssGSEA and risk score, we found that the level of many kinds of immune cells in the high-risk group was significantly lower than that in the low-risk group, including monocytic lineage, plasmacytoid dendritic cell, B lineage, T cells, fibroblasts, neutrophils, myeloid dendritic cells, macrophage, natural killer cell, MDSC, and etc. (**Fig 6B and 6C**). The abundance of five immune cells in each risk group was calculated by the TIMER. Compared with the abundance in the low-risk group, the abundance of CD4 T cell, neutrophil and macrophage was significantly reduced in the high-risk group (**S1A Fig).** Among the 8 immune cells analyzed by EPIC, B cell, cancer associated fibroblast, endothelial cell and macrophage showed significantly lower abundance levels in the high-risk group than in the low-risk group (**S1B Fig**). The strength of the association between risk score and the state of immune cells was measured by correlation degree r calculated by Pearson correlation test. Risk score was indeed significantly negatively correlated with the abundance of multiple immune cell infiltration, including monocytic lineage, endothelial cells, neutrophils, B lineage, fibroblasts and myeloid dendritic cells, etc. (**Fig 6D, S2 Fig**). Moreover, the Pearson correlation test also showed that risk score was significantly negatively linked with the signal pathways regulating immunity, such as cytokine-cytokine receptor interaction, JAK-STAT signaling pathway, complement and coagulation cascade, FC γ R mediated phagocytosis (**Fig 6E**).

### Construction and performance evaluation of optimization model based on risk assessment system

**Fig 7:**
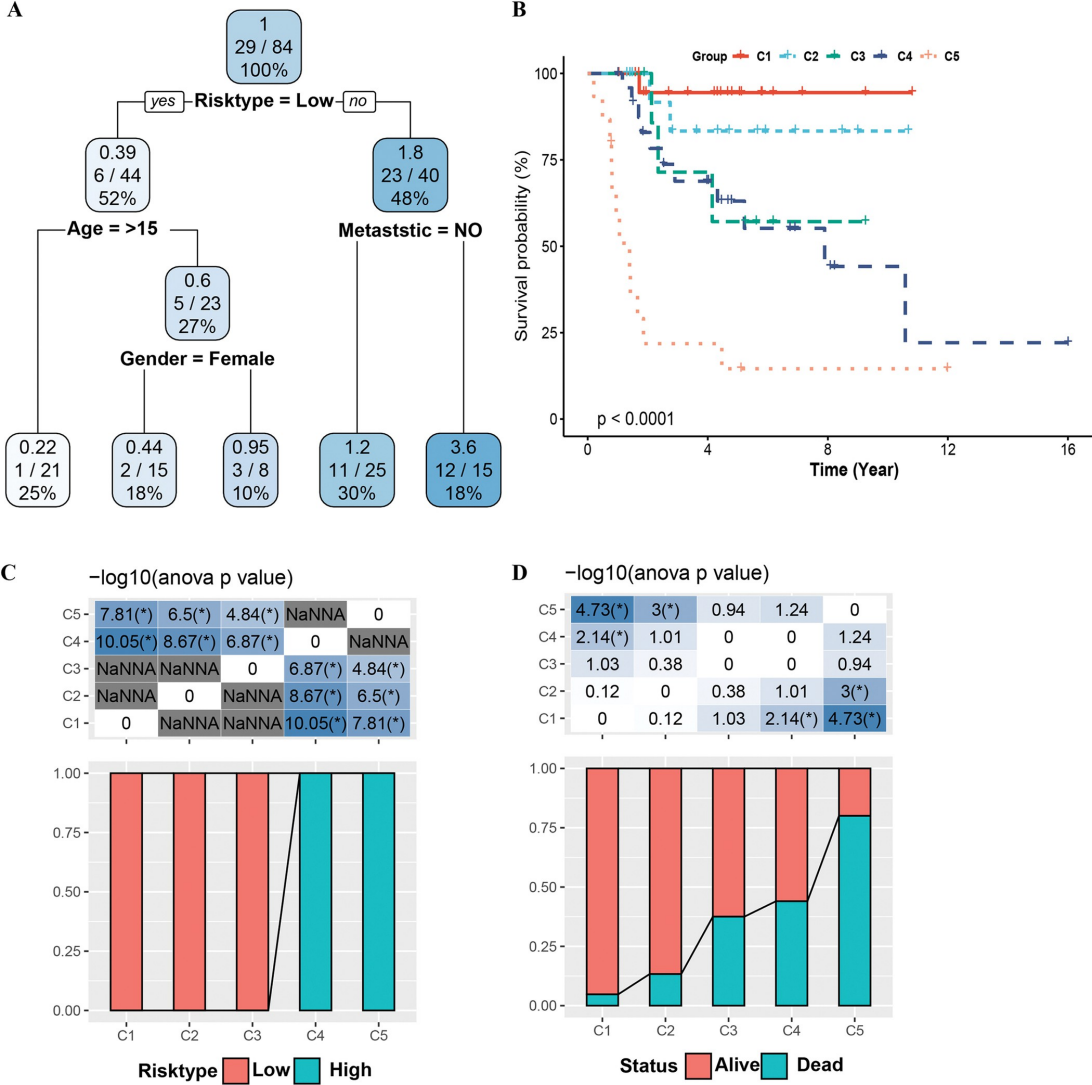
Construction of a decision tree. A: The decision tree classifier model is constructed according to the age, gender, metastatic and risk assessment system of the samples in TARGET. B: OS differences of five clusters divided by the decision tree. C: The distribution of five clusters divided by decision tree in high- and low-risk groups. D: Survival status statistics in the five clusters divided by the decision tree.

**Fig 8:**
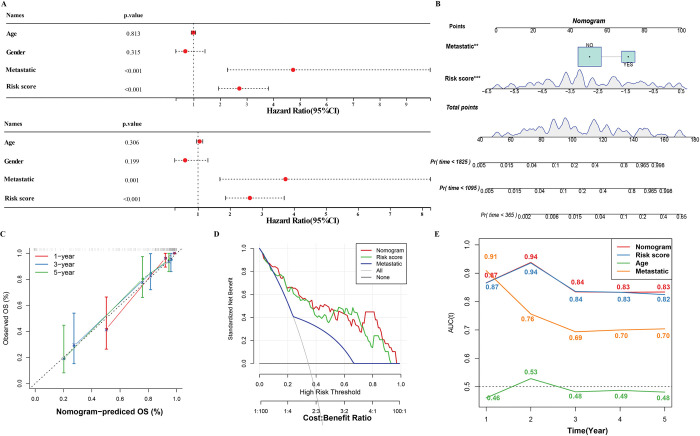
Construction and evaluation of a nomogram based on risk assessment system. A: Univariate COX regression and multivariate COX regression were used to analyze the clinical variables and risk score of TARGET. B: Nomogram integrating risk score and metastatic. C: The calibration curves of nomogram. D: Predictive net benefit of the nomogram, risk assessment system and metastatic. E: Comparing the performance of nomogram, risk assessment system, age, and metastatic osteosarcoma OS by comparing AUC (The AUC value of each indicator for 1–5 years is represented by the indicator’s representative color).

We layered samples and optimized the risk assessment system more accurately by building a decision tree and nomogram. According to the age, gender, metastatic and risk assessment system of the samples in TARGET, recursive partitioning analysis was conducted, and a decision tree was obtained, which formed 4 nodes and 5 subgroups (**Fig 7A**). We detected statistical differences in OS among five subgroups (**Fig 7B**). It was very obvious that C2 and C3 were members of the low-risk group, and C4 and C5 were members of the high-risk group (**Fig 7C**). And from C1 to C5, the proportion of death samples in the group increased with the increase of the cluster number (**Fig 7D**). Metastasis and risk score was an independent variable for predicting OS of osteosarcoma (**Fig 8A**). Therefore, these independent variables are integrated together to build a nomogram (**Fig 8B**). The calibration curve showed that the OS predicted by nomogram was very close to the actual situation at different time points (**Fig 8C**). The DCA showed that in predicting OS, the risk assessment system and nomogram brought more net benefits than the independent prognostic variable metastatic, and also obtained the highest AUC values > 0.6 from 1 to 5yeras (**Fig 8D and 8E**).

## Discussion

There is growing evidence that bone biology is particularly affected by the regulation of redox balance [39]. In healthy tissues, oxidative stress is involved in the maintenance of bone remodeling and the development of bone malignant tumors as well as a poor prognosis [40]. At present, a new strategy for the treatment of osteosarcoma called cold air ionomer based on the mechanism of oxidative stress has been developed [41]. And the survival factors and adaptive pathways for oxidative stress are being explored as targets for cancer treatment and intervention [42]. However, due to the heterogeneity of pathology, the development of therapeutic targets in OS is very complex, which limits the effectiveness of treatment and is conducive to tumor recurrence and drug resistance [4]. The classification of osteosarcoma based on molecular subtypes provides an opportunity for effective treatment of the disease [43]. In this study, according to 45 OSRGs with statistical significance for the prognosis of osteosarcoma, we divided osteosarcoma into two molecular subtypes. Through LASSO and COX regression analysis of DEGs between two molecular subtypes, an independent and prognostic risk assessment system for osteosarcoma was established.

Because osteosarcoma is a complex disease that interacts with its microenvironment and immune system [44], these aspects should not be ignored in the study of this disease. Immune cells, such as T cells, dendritic cells, and macrophages, are the main constitutes of the immunosuppressive TIME from the ROS-induced inflammation [45, 46]. Between the two subgroups, TIME of C2 with better prognosis had more degree of immune infiltration and were more likely to receive ICB therapy. A recently published paper revealed that ICB responders are characterized by CD8+ central memory T cell accumulation [47]. Consistently, we found high proportions of CD8+ central memory T cell in C2 group. What’s more, gastric cancer patients with response to anti PD-1 treatment had high CYT score [48]. High IFN- γ score and T cell inflamed GEP score are suggested to be favorable prognostic indicators for anti-PD-1/PD-L1 [49] or chemotherapeutics therapy [50]. These three high scores in C2 cluster further supported the conclusion that C2 had more possibility for PD-1 checkpoint blockade treatment. Collectively, these results explained the reason for osteosarcoma patients in C2’s better survival time and future treating strategy.

Considering the heterogeneity between molecular subtypes defined by specific oxidative stress genes, we identified the DEGs between the two molecular subtypes and deduced a risk assessment system composing of ZYX, GJA5, GAL, GRAMD1B, and CKMT2 for the prognosis of osteosarcoma. Many of the genes in the risk assessment system we have created have biological significance in the progression of cancer and are related to prognosis. Zyxin (ZYX) is a LIM domain protein found in cytoplasm and nucleus, which has been shown to be involved in apoptosis, migration and invasion of cancer [51, 52], and has potential as a prognostic marker for colorectal cancer 30697742. ZXY was selected a protective gene, which was in line with the findings that overexpressed ZXY could repress the progression of osteosarcoma through Rap1-mediated suppression of the MEK/ERK signaling pathway [53]. GJA5 was identified as one of OS related genes for hepatocellular carcinoma patients, who received transarterial chemoembolization treatment [54]. Nonetheless, no reports of this gene have been found in studies related to osteosarcoma. Galectins (GAL) are a family of endogenous glycan-binding proteins, among which, GAL1, GAL3 and GAL9 can be seen in many types of cancer progression and is considered to be a promising molecular target for the development of cancer therapeutic drugs [55, 56]. Underexpressed GAL1 was reported to inhibit the growth and invasion of osteosarcoma cells through depressing the MAPK/ERK pathway [57], and the inhibition of GAL3 has a therapeutic efficacy of Semliki Forest virus in pediatric osteosarcoma [58]. GRAMD1B inhibits cell migration by negatively regulating JAK / STAT and AKT signal transduction in breast cancer [59]. This gene was firstly discovered in an osteosarcoma model. CKMT2 was selected as a hypoxia-immune-related microenvironment prognostic gene for osteosarcoma [60] as a risk gene, which is consistent with our result. These findings provided evidence for genes in the risk assessment model as prognostic indicators of osteosarcoma.

There were some limitations in this study. First, the small number of OSRGs were obtained and the small number of patients were studied, as well as the lack of some key clinical information, inevitably limits the conclusion. Second, the conclusions were only based on bioinformatics analysis in two retrospective cohorts, and further experimental and clinical validation of our findings are warranted. Third, the internal molecular regulation mechanism of the risk assessment system was not considered, and further research is needed.

## Conclusions

Overall, this study defined two osteosarcoma molecular subtypes. Wherein, C2 cluster displayed prolonged survival time and was more likely to benefit from PD-1 immunotherapy. A risk model composing of ZYX, GJA5, GAL, GRAMD1B, and CKMT2 was established for assessing osteosarcoma prognosis. Patients in low risk group had favorable OS and high degree of immune infiltration. Finally, a nomogram was also designed for future clinical practice. The oxidative stress-related subtypes defined in our study may help reveal the interaction between oxidative stress and the immune microenvironment, and its associated risk models and hub genes have potential implications for future clinical management and precision therapy.

## Supporting information

S1 Fig The abundance of immune cells in each risk group. A: The abundance of five immune cells in each risk group was calculated by the TIMER. B: Abundance levels of the eight immune cells analyzed by EPIC in each risk group.
(PDF)

S2 Fig Correlation between abundance levels of immune cells analyzed by TIMER and EPIC and risk score. (PDF)

## Abbreviation

OSRG: oxdative stress related-gene
LASSO: least absolute shrinkage and selection operator
DEG: differentially expressed gene
TIME: tumor immune microenvironment
OS: overall survival
ROS: reactive oxygen species
TARGET: Therapeutically Applicable Research to Generate Effective Treatments
MsigDB: Molecular Signatures Database
ESTIMATE: Estimation of STromal and Immune cells in Malignant Tumours using Expression data
MCP-counter: Microenvironment Cell Populations-counter
PD-1: programmed death-1
Th1: type 1 helper T
GEP: gene expression profile
GSEA: gene set enrichment analysis
FDR: false discovery rate
Kaplan-: Meier curve, K-M curve
DCA: decision curve analysis
MDSC: myeloid-derived suppressor cell
ZYX: Zyxin
GAL: Galectin
