# Supplementary figures and images for: Construction of a 5-gene prognostic signature based on oxidative stress related genes for predicting prognosis in osteosarcoma
Source: PLoS One. 2023 Dec 1;18(12):e0295364. doi: 10.1371/journal.pone.0295364 (PMC10691720; doi:10.1371/journal.pone.0295364)

group High Low

A

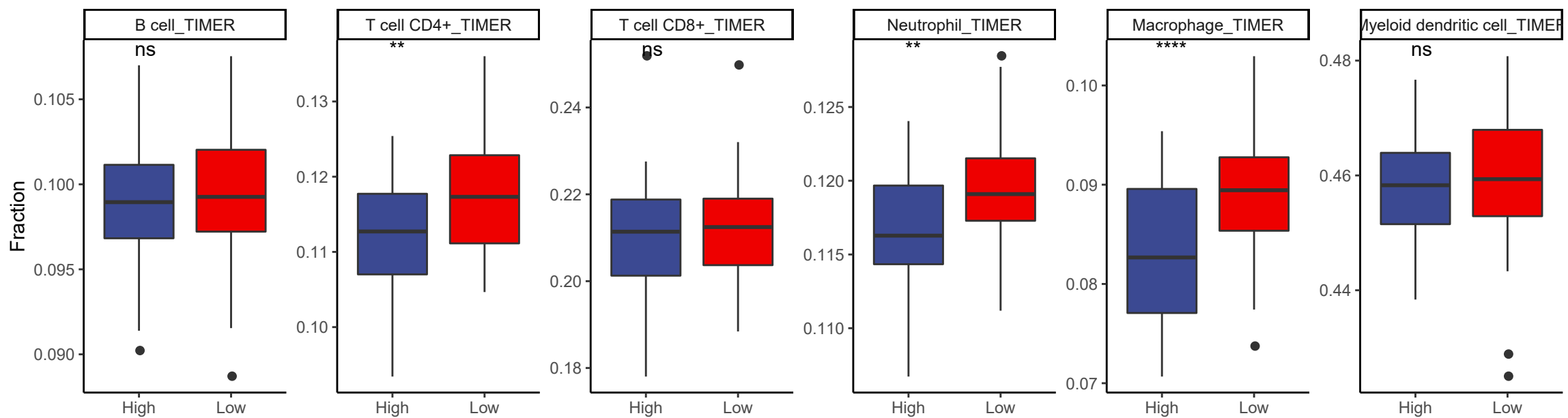

B

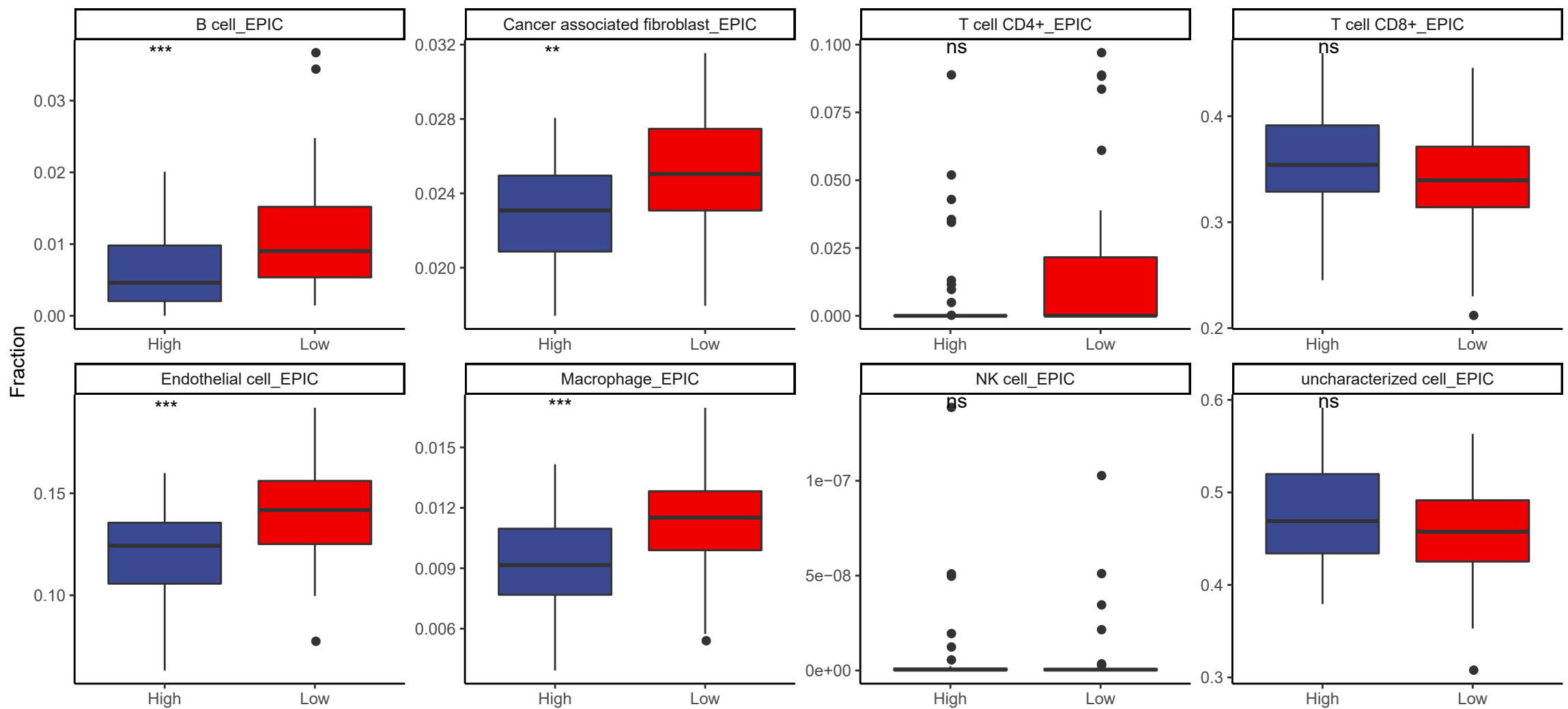

Supplement: S1 Fig — A: The abundance of five immune cells in each risk group was calculated by the TIMER. B: Abundance levels of the eight immune cells analyzed by EPIC in each risk group. (PDF) [file pone.0295364.s001.pdf]

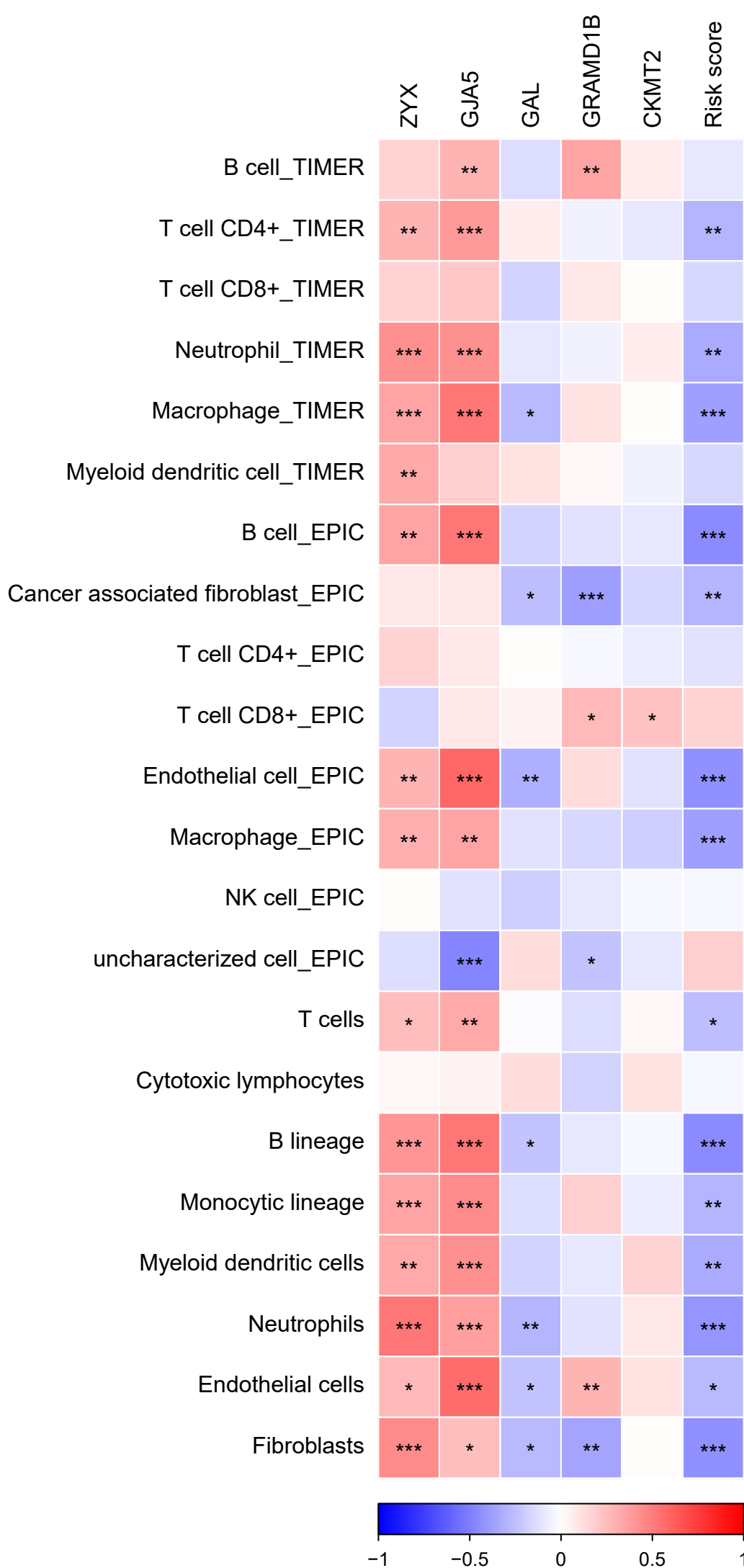

Supplement: S2 Fig — (PDF) [file pone.0295364.s002.pdf]
